# A rare *de novo* contiguous 15q11.1-q13.3 duplication with tetrasomy (CN=4) and adjacent trisomy (CN=3) associated with severe global developmental delay, autism spectrum disorder, and subclinical epileptiform discharges: a case report and literature review

**DOI:** 10.3389/fgene.2026.1953023

**Published:** 2026-09-09

**Authors:** Xinyu Zhou, HongYan Xie, Haoyan Yu, Zhiyuan Zhang, Yangfan Qi, Kai Jiang, Shuangzhu Lin

**Affiliations:** 1 Changchun University of Chinese Medicine, Changchun, China; 2 Diagnosis and Treatment Center for Children, The Affiliated Hospital of Changchun University of Chinese Medicine, Changchun, China

**Keywords:** 15q11.2-q13 duplication syndrome, autism spectrum disorder (ASD), Dup15q syndrome, gene dosage effect, global developmental delay (GDD), subclinical epileptiform discharges

## Abstract

**Background:**

15q11.2–q13 duplication syndrome (Dup15q; OMIM #608636) is a rare neurodevelopmental disorder. While interstitial duplications (copy number = 3) are relatively well characterized, contiguous rearrangements comprising both tetrasomic (copy number = 4) and adjacent trisomic (copy number = 3) segments are rare. We report a Chinese girl with a *de novo* contiguous 15q11.1–q13.3 duplication, whose underlying genetic diagnosis was initially delayed because her early neurodevelopmental abnormalities were partly attributed to prematurity.

**Case Presentation:**

The patient was born at 35+2 weeks of gestation and presented with global developmental delay, severe autism spectrum disorder (CARS: 44), and profound cognitive and language impairment (GQ 30, GMQ 57 at 2 years 4 months). Brain MRI demonstrated delayed myelination, and magnetic resonance spectroscopy showed metabolic abnormalities in the left frontal lobe. Video-electroencephalography at 3 years of age revealed sleep-activated frontocentral epileptiform discharges without clinical seizures. Trio whole-genome sequencing with copy number variation analysis identified a *de novo* contiguous duplication consisting of a ∼10.34 Mb tetrasomic segment (15q11.1–q13.2; CN = 4) and an adjacent ∼2.40 Mb trisomic segment (15q13.2–q13.3; CN = 3). Despite long-term rehabilitation, the patient remained nonverbal and exhibited persistent severe neurodevelopmental impairment.

**Conclusion:**

This case expands the genomic spectrum of complex proximal 15q rearrangements and highlights the value of high-resolution genomic testing for resolving complex neurodevelopmental disorders. It also emphasizes that severe genetic etiologies may be overlooked when developmental abnormalities are initially attributed to prematurity. These findings support early comprehensive genomic evaluation in children with profound developmental delay, autism spectrum disorder, or persistent developmental impairment despite conventional rehabilitation.

## 1 Introduction

The proximal long arm of chromosome 15 (15q11.2–q13) is one of the most unstable and structurally complex regions in the human genome. Flanked by low-copy repeats (LCRs), designated as breakpoints (BP1–BP5), this region is highly susceptible to non-allelic homologous recombination (NAHR), resulting in recurrent microdeletions and microduplications (Christian et al., 1999; Lusk et al., 2016). Among these, 15q11.2–q13 duplication syndrome (Dup15q; OMIM #608636) is a well-recognized neurodevelopmental disorder characterized by global developmental delay (GDD), autism spectrum disorder (ASD), hypotonia, intellectual disability, speech impairment, and an increased risk of epilepsy (Lusk et al., 2016; Battagl and ia, 2008). Dup15q syndrome is subject to genomic imprinting. While paternally inherited duplications are often associated with a normal or only mildly affected phenotype, maternally derived or *de novo* duplications are associated with substantially greater clinical severity and a markedly higher risk of neurodevelopmental impairment (Lusk et al., 2016; Urraca et al., 2013).

The clinical severity of Dup15q syndrome is highly heterogeneous and is closely associated with gene dosage and the structural configuration of the duplicated chromosome. Interstitial duplications typically result in three genomic copies (trisomy 15q), whereas maternal isodicentric chromosome 15 [idic(15)] or complex maternal interstitial triplications result in four copies (tetrasomy 15q), which are generally associated with more severe intellectual disability, autism spectrum disorder, and treatment-resistant epilepsy (Lusk et al., 2016; Battagl and ia, 2008; Al Ageeli et al., 2014). At the molecular level, these phenotypic differences are thought to result primarily from increased dosage of multiple dosage-sensitive genes within the Prader-Willi/Angelman syndrome critical region, particularly UBE3A, which has been implicated in synaptic development and autism spectrum disorder, and the GABAA receptor gene cluster (GABRB3, GABRA5, and GABRG3), which has been associated with altered inhibitory neurotransmission, electroencephalographic (EEG) abnormalities, and epilepsy (Greer et al., 2010; Hogart et al., 2010). Furthermore, duplications extending into the adjacent 15q13.2–q13.3 region, encompassing the nicotinic acetylcholine receptor gene CHRNA7, have also been proposed to modify the clinical phenotype and may further contribute to neurodevelopmental and neuropsychiatric manifestations (Gillentine and Schaaf, 2015).

In pediatric practice, identifying the underlying genetic etiology of GDD and ASD may be complicated by coexisting perinatal factors, particularly prematurity. Early developmental delay, motor impairment, and nonspecific neuroimaging findings (e.g., delayed myelination) in preterm infants may initially be attributed to the sequelae of prematurity, thereby delaying timely genetic evaluation and definitive molecular diagnosis (Srivastava et al., 2019).

Here, we report a pediatric patient with a history of prematurity who presented with severe neurodevelopmental impairment and subclinical epileptiform activity. High-resolution genomic analysis identified a rare *de novo* contiguous 15q11.1–q13.3 duplication comprising an approximately 10.34 Mb tetrasomic segment (CN = 4) and an adjacent 2.40 Mb trisomic segment (CN = 3). This unusual genomic copy number profile provides a valuable opportunity to investigate the potential contribution of adjacent dosage-sensitive genes, including UBE3A, the GABAA receptor gene cluster, and CHRNA7, to the observed clinical phenotype. Moreover, this case highlights the importance of early comprehensive genomic evaluation in premature children with persistent and severe neurodevelopmental impairment that cannot be fully explained by prematurity alone. By presenting this case together with a systematic review of the literature, we aim to expand the clinical and molecular spectrum of complex proximal 15q duplications, summarize the associated genotype–phenotype correlations, and improve the recognition and genetic diagnosis of this rare disorder.

## 2 Methods

### 2.1 Ethics statement

This study was approved by the Ethics Committee of the Affiliated Hospital of Changchun University of Chinese Medicine and was conducted in accordance with the principles of the Declaration of Helsinki. Written informed consent was obtained from the patient’s legal guardians for participation in this study and for the publication of this case report, including the accompanying clinical data, pedigree, neurophysiological findings, and genomic results (World Medical Association, 2013).

### 2.2 Genetic analysis

To investigate the molecular basis of the proband’s phenotype, Trio-based copy number variation sequencing (Trio-CNV-seq) was performed using peripheral blood samples. Genomic DNA was extracted, fragmented, library-prepared, and sequenced on the Illumina platform (Illumina, San Diego, CA, United States of America) according to the manufacturer’s protocol. Sequencing reads were aligned to the human reference genome (GRCh37/hg19). A bioinformatics pipeline based on read-depth analysis was employed to detect submicroscopic chromosomal abnormalities, determine coordinates, and estimate absolute copy numbers. Pathogenicity was classified according to the joint ACMG and ClinGen technical standards (Riggs et al., 2020).

### 2.3 Systematic literature search and comparative analysis

A systematic literature search was conducted across the PubMed, Web of Science, and CNKI databases up to March 2026. Search strategies combined keywords including “15q11.2–q13 duplication syndrome,” “Dup15q syndrome,” “interstitial 15q duplication,” “15q tetrasomy,” “CHRNA7 duplication,” and “developmental delay.” Studies were included if they presented molecularly confirmed copy number gains (CN ≥ 3) involving the 15q11–q13 and/or 15q13.3 regions, alongside detailed clinical, behavioral, or electrophysiological (EEG) phenotypes. Clinical, electrophysiological, and genomic data from ten previously published cases (Urraca et al., 2013; Hogart et al., 2010; Lu et al., 2020; Fakir et al., 2026; Bundey et al., 1994; Orrico et al., 2009; Park et al., 2013; Liu et al., 2026; Battaglia et al., 2010), alongside the present case, were summarized in Table 1.

**TABLE 1:** Clinical and genetic characteristics of the present case compared with previously reported cases of 15q11-q13 duplications/triplications.

Study/Case no.	Gender/Age	Chromosomal region & Size (Mb)	Copy number & variant type	Inheritance	GDD/ID	ASD	Motor deficits & hypotonia	Epilepsy/EEG Abnormality	Brain MRI/MRS findings
Present case	F/3years 5 m	15q11.1-q13.2 (∼10.34 Mb) & 15q13.2-q13.3 (∼2.40 Mb)	CN = 4 (double duplication) & CN = 3 (single duplication)	*De novo*	Yes (severe, griffiths GQ = 30)	Yes (CARS = 44)	Yes (Peabody GMQ = 57, hypotonia, clumsy gait, tip-toeing)	No clinical seizures; EEG showed bilateral frontocentral spikes during sleep	Periventricular signals (myelination lag); MRS showed elevated NAA/Cr and decreased Cho/Cr
Lu et al., 2020	M/11 m (followed to 19 m)	15q11.1-q13.2 (∼10.16 Mb) & 15q13.2-q13.3 (∼1.84 Mb)	CN ≈ 4 (3.96 copies, sSMC) & CN ≈ 3 (3.03 copies)	*De novo*	Yes (global developmental delay)	Yes (autistic tendencies, poor response to verbal cues)	Yes (hypotonia, sat with support at 9 m, stood alone at 19 m, unable to walk by 2years)	No clinical seizures; EEG showed conductive β*β* rhythm and sharpened frontal waves	Normal brain MRI
Zineb et al., 2026	F/8 y	15q11.2-q13.3 (∼9.0 Mb)	CN = 3 (Heterozygous duplication)	Unknown	Yes (global developmental delay, intellectual disability)	Yes (ASD, aggressiveness, hyperactivity)	Yes (motor delay, walking on tiptoes)	No clinical seizures; normal EEG	ND
Bundey et al., 1994	M/2 years (followed to 14years)	15q11-q13 (interstitial duplication)	CN = 3 (duplication of *GABRA5* & *GABRB3*)	Maternal	Yes (severe mental retardation)	Yes (autistic syndrome, ritualistic behaviors, echolalia)	Yes (hypotonia at 4 m, sat at 10 m, walked at 23 m, ataxic gait)	Yes (tonic-clonic seizures starting at 18 m, grand mal attacks); EEG was normal	ND
Orrico et al., 2009	F/33 y	15q11.2-q13.1 (∼4.0 Mb)	CN = 3 (microduplication)	*De novo*, maternal	Yes (severe mental retardation, loss of speech at 13years)	Yes (autistic features, social reduction, stereotypic hand-washing)	Yes (hypotonia, poor motor skills, coordination decline)	Yes (Intractable epilepsy, tonic & partial seizures; late-onset Lennox-gastaut syndrome); EEG: Spike-waves	Hypoplasia of corpus callosum, moderate cortical atrophy
Park et al., 2013	M/9 m (followed to 19 m)	15q11-q13 (idic(15)(q12))	CN = 4 (tetrasomy 15q via idic(15))	*De novo*	Yes (developmental delay, cognitive and language lags)	No (CARS = 15, no autistic behaviors up to 19 m)	Yes (Diffuse hypotonia, sat with support at 9 m, stood at 19 m)	No clinical seizures; normal EEG	Normal brain MRI (at 4 m)
Zou et al., 2020 (patient 1)	M/3 y	15q11.2-q13.1 (∼5.5 Mb)	CN = 3 (interstitial duplication)	Maternal	Yes (developmental delay, speech delay)	Yes (ASD traits)	Yes (hypotonia, motor delays)	No clinical seizures; EEG: Normal	Normal brain MRI
Zou et al., 2020 (patient 5)	F/5 y	15q11-q13 (idic(15))	CN = 4 (tetrasomy 15q via idic(15))	*De novo*	Yes (profound intellectual disability)	Yes (severe ASD)	Yes (severe hypotonia, unable to sit or walk)	Yes (Infantile spasms/epilepsy, intractable); EEG: Hypsarrhythmia	Moderate cerebral atrophy
Battaglia et al., 2010 (case 1)	F/4 y	15q11.2-q13.1 (idic(15))	CN = 4 (tetrasomy 15q via idic(15))	*De novo*	Yes (severe developmental delay, absent speech)	Yes (autistic features)	Yes (severe hypotonia, unable to walk)	Yes (Infantile spasms/epilepsy); EEG: Multifocal spikes	Normal brain MRI
Urraca et al., 2013 (case 3)	M/6 y	15q11.2-q13 (interstitial duplication)	CN = 3 (interstitial duplication)	Maternal	Yes (mild intellectual disability)	Yes (ASD, stereotypic behaviors)	Yes (motor coordination deficits, clumsy gait)	No clinical seizures; EEG: Characteristic beta-warmth rhythms	Normal brain MRI
Hogart et al., 2010 (case 1)	F/5 y	15q11-q13 (interstitial duplication)	CN = 3 (interstitial duplication)	Maternal	Yes (developmental delay, developmental regression)	Yes (autistic features, hyperactivity)	Yes (hypotonia, motor delay)	No clinical seizures; EEG: Normal	Normal brain MRI

F, female; M, male; y, years; m, months; CN, copy number; idic(15), isodicentric chromosome 15; sSMC, small supernumerary marker chromosome; GDD, global developmental delay; ID, intellectual disability; ASD, autism spectrum disorder; EEG, electroencephalogram; MRI, magnetic resonance imaging; MRS, magnetic resonance spectroscopy; ND, not determined/not reported.

## 3 Case Presentation

### 3.1 Patient history and perinatal course

**FIGURE 1:**
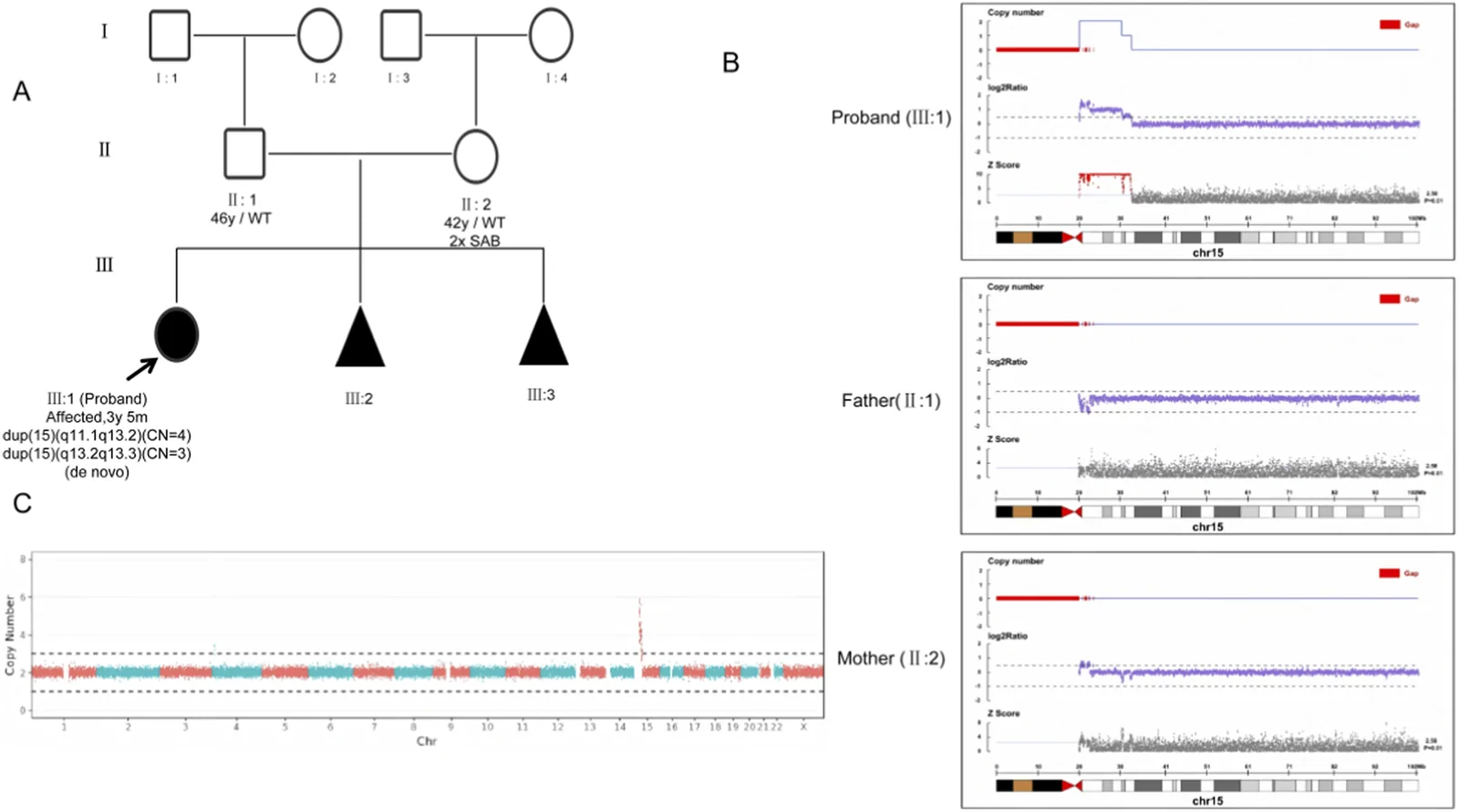
Pedigree, whole-genome landscape, and locus-specific genomic analysis of the family. **(A)** Three-generation pedigree of the family. The affected female proband (III:1) is indicated by a blackened circle and an arrow. Paternal (II:1) and maternal (II:2) parents are phenotypically normal and carry wild-type (WT) alleles. The mother has a clinical history of two spontaneous abortions (SAB; II:2 and II:3) at approximately 2 months of gestation. **(B)** Chromosome 15 CNV-seq profiles. The proband’s profile (top) reveals a *de novo* ∼10.34 Mb double duplication at chromosome 15q11.1-q13.2 (copy number = 4) and an adjacent ∼2.40 Mb single duplication at 15q13.2-q13.3 (copy number = 3). Profiles of the father (middle) and mother (bottom) demonstrate a normal diploid copy number (copy number = 2) at the corresponding loci, confirming the *de novo* origin of the proband’s variants. **(C)** Whole-genome CNV-seq panoramic profile of the proband across chromosomes 1–22 and X. A single distinct signal spike is observed exclusively on chromosome 15 (red dots), indicating that the 15q duplication is the sole significant copy number aberration across the entire genome.

The female patient was born on 27 December 2022, to healthy, non-consanguineous young parents. This was the mother’s third pregnancy and first live birth (gravida 3, para 1; G3P1). Her obstetric history was notable for two unexplained spontaneous abortions during the first trimester, both occurring at approximately 2 months of gestation (Figure 1A). The patient was delivered by Cesarean section at 35+2 weeks of gestation because of obstetric indications. Her birth weight was 3.1 kg, classifying her as a large-for-gestational-age (LGA) infant,and she showed no evidence of neonatal asphyxia or need for resuscitation after birth. The family history was unremarkable for neurodevelopmental disorders, epilepsy, or known genetic diseases.

### 3.2 Clinical presentation and neurodevelopmental evaluations

The patient exhibited significant developmental delay from early infancy, achieving head control at 3 months, independent sitting at 12 months, and independent walking with an unsteady ataxic gait at 30 months. Speech development was severely delayed; she began non-purposeful babbling at 6 months but failed to develop expressive speech. Standardized neurodevelopmental and behavioral evaluations performed at 2 years and 4 months of age confirmed severe global developmental delay (GDD) and severe autism spectrum disorder (ASD) (detailed in Table 2).

**TABLE 2:** Developmental and behavioral evaluation results of the proband at 2 years and 4 months of age.

Evaluation Scale	Domain/Subscale	Raw score/Equivalent age	Developmental quotient (DQ)/Score	Clinical interpretation
GMDS	Global/Total	—	GQ = 30	Profound global developmental delay
​	A: Gross motor	12.5 months	DQ = 44	Severe delay
​	B: Personal-Social	9.5 months	DQ = 33	Severe delay
​	C: Hearing & language	6.5 months	DQ = 23	Profound delay
​	D: Eye-Hand Coordination	7.0 months	DQ = 25	Severe delay
​	E: Performance	7.5 months	DQ = 26	Severe delay
PDMS-2	Gross motor	—	GMQ = 57	Severe motor delay (<1st percentile)
CARS	Behavioral severity	—	Total score = 44	Severe autism spectrum disorder
M-CHAT-R	ASD screening	—	9 positive items (4 core)	High risk for ASD (Positive)
SM Scale	Social Life ability	—	Standard score = 8	Borderline-to-mild Social abnormality

Abbreviations: GMDS, griffiths mental development scales; GQ, developmental quotient; PDMS-2, peabody developmental motor scales; GMQ, gross motor quotient; CARS, childhood autism rating scale; ASD, autism spectrum disorder; M-CHAT-R, modified checklist for autism in toddlers, Revised; SM, scale, Social Life Ability Scale.

Upon admission at 3 years of age, her body weight was 12.0 kg (approx. −1.0 SD) and her height was 93.5 cm (approx. −0.5 SD), both within normal limits. Neurological examination showed bilateral equinus gait (toe walking) and muscle weakness restricted to the lower extremities, with preserved upper limb strength. Behavioral evaluation demonstrated poor eye contact, absence of expressive speech, poor response to her name, inability to follow simple commands, and a persistent fascination with spinning objects (Figure 3).

### 3.3 Electrophysiological and neuroimaging investigations

Brain magnetic resonance imaging (MRI) performed at 2 years and 4 months of age demonstrated nonspecific T1-and T2-weighted signal abnormalities adjacent to the anterior and posterior horns of the bilateral lateral ventricles, consistent with delayed myelination. Magnetic resonance spectroscopy (MRS) of the left frontal lobe showed an elevated N-acetylaspartate/creatine (NAA/Cr) ratio of 1.82 and a decreased choline/creatine (Cho/Cr) ratio of 1.05. Routine laboratory investigations, including complete blood count, blood ammonia, homocysteine, thyroid function tests, and blood and urine metabolic screening, revealed no significant abnormalities. Serum trace element analysis showed calcium, magnesium, iron, and zinc levels of 66.1 mg/L, 40.1 mg/L, 443.3 mg/L, and 5.0 mg/L, respectively.

**FIGURE 2:**
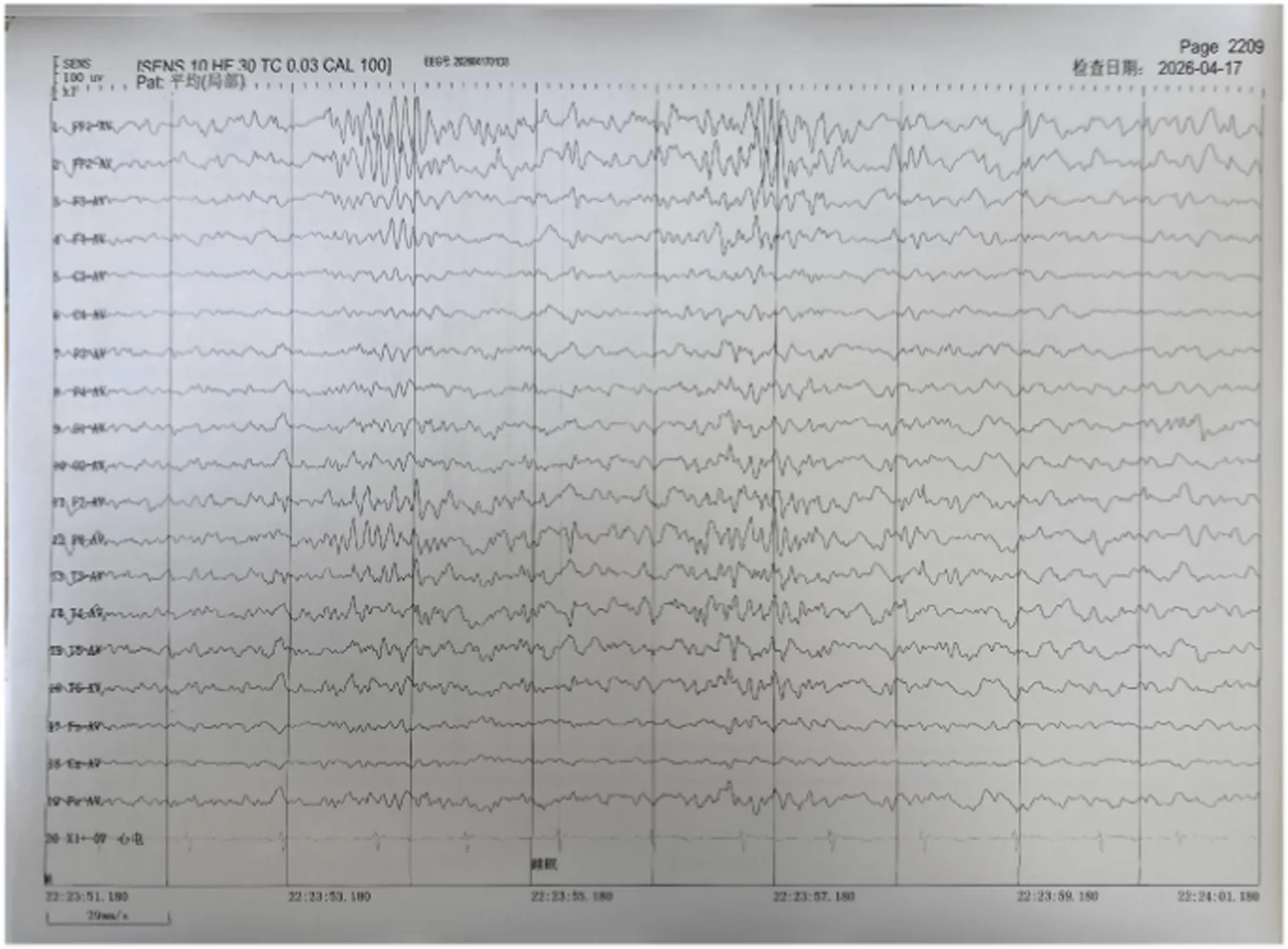
Representative sleep-phase video-EEG recording of the proband at 3 years of age. The 6-h video-EEG tracing (Page 2209) demonstrates a sleep-background interspersed with paroxysmal, synchronous, and symmetrical bursts of high-voltage spike-and-slow wave complexes. These epileptiform discharges are predominantly localized in the bilateral frontocentral regions, as most clearly delineated in the upper frontal channels (FP1-AV, FP2-AV, F3-AV, and F4-AV). No clinical convulsions, tonic events, or corresponding behavioral symptoms were captured during these electrographic paroxysms, confirming the subclinical nature of the epileptiform activity. (Sensitivity: 100 μV, High-frequency filter: 30 Hz, Time constant: 0.03 s, Calibration: 100 μV, Paper speed: 29 mm/s).

At 3 years of age, a 6-h video-electroencephalogram (video-EEG) was performed. The awake background activity consisted of a poorly regulated 9-Hz alpha rhythm. During sleep, frequent synchronous high-voltage spike-and-slow-wave discharges were observed predominantly over the bilateral frontocentral regions (Figure 2). No clinical seizures or behavioral changes were observed during these electrographic discharges, indicating subclinical epileptiform activity.

**FIGURE 3:**
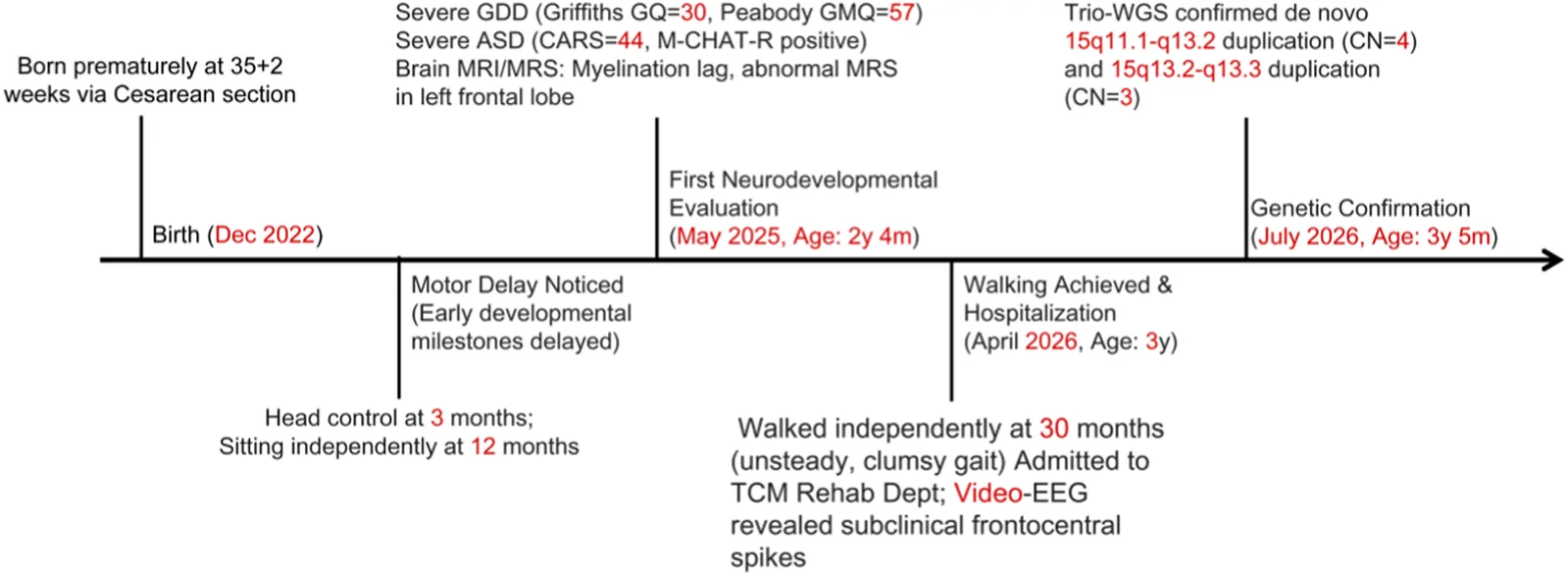
Timeline of the clinical course and diagnostic odyssey of the proband. Birth (December 2022): Premature birth at 35+2 weeks of gestation. Early Infancy: Recognition of motor milestones delays, including head control at 3 months and independent sitting at 12 months. Comprehensive Evaluation (May 2025, 2years 4 m): Formal diagnosis of severe global developmental delay (GDD) and severe autism spectrum disorder (ASD), accompanied by brain MRI/MRS indicating delayed myelination. Rehabilitation & Electrographic Assessment (April 2026, 3years): Independent walking achieved at 30 months; subsequent admission for traditional Chinese medicine (TCM) rehabilitation, where video-EEG identified subclinical frontocentral spikes. Genetic Confirmation (July 2026, 3years 5 m): Definitive diagnosis achieved via Trio-whole genome sequencing (Trio-WGS), revealing a complex *de novo* duplication on chromosome 15.

### 3.4 Genomic analysis

At 3 years and 5 months of age, Trio whole-genome sequencing (Trio-WGS) combined with CNV-seq was performed to investigate the underlying molecular etiology. Conventional G-banding demonstrated a normal female karyotype (46,XX). However, high-resolution CNV-seq identified a rare *de novo* complex rearrangement involving chromosome 15 (Figures 1B,C):

1. A ∼10.34 Mb tetrasomic duplication involving chr15:20,000,001–30,336,944 (15q11.1–q13.2), encompassing multiple dosage-sensitive genes, including *UBE3A*, *GABRB3*, *GABRA5*, and *GABRG3* (Table 3).
2. An adjacent ∼2.40 Mb trisomic duplication involving chr15:30,336,945–32,737,944 (15q13.2–q13.3), encompassing *CHRNA7* (Table 3).

**TABLE 3:** Key dosage-sensitive genes within the duplicated 15q11-q13 and 15q13.3 regions and their clinical relevance.

Gene symbol	Chromosomal band	Physiological function	Contribution to the Dup15q phenotype
*UBE3A*	15q11.2	Encodes ubiquitin protein ligase E3A; exhibits maternal-specific expression in neurons; crucial for synaptic pruning and dendritic spine development	Overexpression of maternal UBE3A is the primary driver of autism spectrum disorder (ASD) features, severe language deficits, and cognitive impairment
*GABRB3*	15q12	Encodes the β3 subunit of the GABAA receptor; mediates inhibitory neurotransmission in the central nervous system	Increased dosage disrupts the excitation/inhibition (E/I) balance, contributing to EEG abnormalities (e.g., beta-warmth, spike-waves), muscle hypotonia, and epilepsy
*GABRA5*	15q12	Encodes the α5 subunit of the GABAA receptor; involved in synaptic and extrasynaptic tonic inhibition in the hippocampus.	Synergistically impairs GABAergic transmission with GABRB3, associated with cognitive dysfunction, motor coordination deficits, and seizure susceptibility
*GABRG3*	15q12	Encodes the γ3 subunit of the GABAA receptor; essential for receptor clustering and synaptic localization	Linked to altered behavioral phenotypes, sensory processing sensitivity, and neurodevelopmental delays
*CHRNA7*	15q13.3	Encodes the α7 subunit of the nicotinic acetylcholine receptor; highly expressed in the brain, modulating synaptic transmission and plasticity	Located in the adjacent 15q13.3 microduplication region; its overexpression is associated with attention deficits, cognitive impairment, schizophrenia, and neuropsychiatric symptoms
*NIPA1*	15q11.2	Encodes magnesium transporter protein; modulates bone morphogenetic protein (BMP) signaling pathway; essential for axonal maintenance	Overexpression may contribute to motor pathway dysfunction, spasticity, and the characteristic abnormal gait (e.g., tip-toe walking)
*HERC2*	15q13.1	Encodes a giant scaffold protein and E3 ubiquitin ligase; interacts with UBE3A and modulates cellular response to DNA damage	Modulates UBE3A activity; homozygous mutations cause mental retardation, while duplication/overexpression is linked to developmental delays and autistic traits
*NDN*	15q11.2	Necdin; a paternally expressed imprinted gene; promotes axonal outgrowth and neuron survival during early development	Involved in the regulation of neurogenesis and hypothalamic development; associated with feeding difficulties in infancy and cognitive delays

GABA, gamma-aminobutyric acid; EEG, electroencephalogram; ASD, autism spectrum disorder; E/I, excitation/inhibition.

UBE3A undergoes maternal-specific imprinting in the brain, meaning only the maternal copy is active in neurons, making maternal duplications highly pathogenic.

CHRNA7 duplication acts as an additive genetic modifier in this patient, potentially exacerbating the cognitive and behavioral phenotypes.

According to the joint technical standards of the American College of Medical Genetics and Genomics (ACMG) and the Clinical Genome Resource (ClinGen) for constitutional copy number variants (Riggs et al., 2020), the genomic findings of the proband were evaluated independently:

1. The ∼10.34 Mb double duplication at 15q11.1–q13.2 was classified as Pathogenic with a cumulative score of 1.15 points based on the following evidence: Section 1A (0 points) for containing multiple protein-coding genes; Section 2A (1.0 point) due to its complete overlap with the established triplosensitive region associated with Dup15q syndrome (ISCA-37404); and Section 5A (0.15 points) as the variant arose *de novo* with confirmed parental wild-type alleles.
2. The adjacent contiguous ∼2.40 Mb single duplication at 15q13.2–q13.3, which includes CHRNA7, was independently classified as a Variant of Uncertain Significance (VUS) with a cumulative score of 0.15 points based on the following criteria: Section 1A (0 points) for containing protein-coding genes; Section 2G (0 points) because it overlaps with the 15q13.3 microduplication, which is currently established as a susceptibility locus with incomplete penetrance and variable expressivity rather than a fully penetrant pathogenic locus; and Section 5A (0.15 points) due to its confirmed *de novo* origin.

Conventional G-banding and parental CNV analysis confirmed that both *de novo* copy number gains occurred on the paternal or maternal allele of the proband.

### 3.5 Therapeutic intervention and follow-up

Following the clinical diagnosis, the patient received comprehensive multidisciplinary rehabilitation, including physical therapy, occupational therapy, sensory integration therapy, and acupuncture. Despite prolonged intervention, only minimal developmental improvement was observed. At the most recent follow-up at 3 years and 5 months of age, rehabilitation therapy had been discontinued by her parents because of the limited clinical benefit. She remained completely nonverbal and continued to exhibit profound developmental delay, severe autism spectrum disorder, persistent motor impairment, and autistic behaviors.

## 4 Discussion

### 4.1 Unique genomic copy number profile and myelination delay: hypothesized roles of CYFIP1 and TUBGCP5

The genomic copy number profile identified in our patient is rare, consisting of a contiguous ∼10.34 Mb tetrasomic duplication of 15q11.1–q13.2 (CN = 4) and an adjacent ∼2.40 Mb trisomic duplication of 15q13.2–q13.3 (CN = 3) (Figure 1) (Lu et al., 2020; Liu et al., 2026). This unusual combination of interstitial tetrasomy and trisomy within a single *de novo* rearrangement expands the molecular spectrum of Dup15q syndrome and provides an opportunity to explore the potential contribution of regional gene dosage to phenotypic variability.

The neuroimaging findings in our patient may be relevant to this distinctive genomic copy number profile. Brain magnetic resonance imaging (MRI) demonstrated delayed myelination adjacent to the bilateral lateral ventricles, accompanied by a reduced choline/creatine (Cho/Cr) ratio on magnetic resonance spectroscopy (MRS). Because choline is considered a marker of membrane phospholipid metabolism and myelin turnover, these findings may reflect impaired white matter maturation (Ross and Sachdev, 2004). Notably, the duplicated BP1–BP2 interval contains *CYFIP1* and *TUBGCP5*, two genes that have been implicated in early brain development and neurodevelopmental disorders (Table 3). *CYFIP1* encodes cytoplasmic FMR1-interacting protein 1, which participates in actin cytoskeleton remodeling and has been implicated in oligodendrocyte differentiation and myelination (Silva, 2019). *TUBGCP5* is involved in microtubule nucleation and cellular cytoskeletal organization (Cox and Butler, 2015). Although direct evidence linking increased dosage of these genes to delayed myelination in Dup15q syndrome remains limited, we hypothesize that their potential overexpression might have contributed to the neuroimaging abnormalities observed in our patient, representing a plausible genotype-phenotype correlation that warrants further functional validation.

### 4.2 Molecular basis of motor and behavioral deficits: potential contributions of *NIPA1* and the *UBE3A*–Arc pathway

One notable clinical feature of our patient was the predominance of motor impairment in the lower extremities, manifested by hypotonia, muscle weakness, bilateral toe walking (equinus gait), and impaired gait, whereas muscle strength in the upper limbs was relatively preserved. This phenotype may be related, at least in part, to increased dosage of *NIPA1* within the duplicated BP1–BP2 interval (Table 3) (Cox and Butler, 2015). *NIPA1* encodes a magnesium transporter involved in neuronal development and axonal maintenance, and pathogenic variants in *NIPA1* are a well-established cause of hereditary spastic paraplegia type 6 (SPG6), a disorder characterized predominantly by corticospinal tract dysfunction affecting the lower extremities (Rainier et al., 2003; Blackstone, 2018). Although the pathogenic consequences of *NIPA1* duplication remain incompletely understood, the four-copy dosage observed in our patient is hypothesized to be a potential modifier contributed to her selective lower-limb motor impairment.

The patient also exhibited severe autism spectrum disorder, profound cognitive impairment (GQ = 30), and an elevated N-acetylaspartate/creatine (NAA/Cr) ratio in the left frontal lobe. N-acetylaspartate (NAA) is widely regarded as a marker of neuronal integrity and neuronal metabolism (Ross and Sachdev, 2004). Among the duplicated genes, *UBE3A* represents one of the most dosage-sensitive candidates implicated in the neurodevelopmental phenotype of Dup15q syndrome (Lusk et al., 2016). Experimental studies have suggested that excessive *UBE3A* expression may enhance the degradation of activity-regulated cytoskeleton-associated protein (Arc), thereby disrupting activity-dependent synaptic plasticity and normal synaptic maturation (Greer et al., 2010). Although the present study did not directly assess *UBE3A* or Arc expression—and no methylation testing was performed to verify the maternal origin of this *de novo* duplication—an increased UBE3A dosage is hypothesized to contribute to the neurodevelopmental phenotype observed in this patient. Likewise, the limited response to conventional rehabilitation may reflect the overall genetic severity of the disorder rather than the demonstrated effects of a single molecular pathway.

### 4.3 The neurophysiological horizon: potential modifying effects of *CHRNA7* on epileptiform activity

At 3 years of age, the patient exhibited frequent sleep-related frontocentral spike-and-slow-wave discharges on video-EEG (Figure 2) despite the absence of clinically apparent seizures. This electroclinical dissociation may be related to the contiguous ∼2.40 Mb trisomic duplication of the 15q13.2–q13.3 region, which encompasses CHRNA7 (Table 3) (Gillentine and Schaaf, 2015). Although no functional receptor studies were performed, it is theoretically plausible that the additional copy of CHRNA7 might partially modulate the imbalance between excitatory and inhibitory neuronal networks during early brain development, potentially contributing to the presence of subclinical epileptiform discharges in the absence of overt seizures. However, this interaction remains highly speculative and requires validation in future expression models (Sinkus et al., 2015).

Nevertheless, previous studies have demonstrated that epilepsy frequently develops with increasing age in patients with maternal Dup15q syndrome, particularly in those carrying high-copy-number rearrangements (Table 1) (Lusk et al., 2016; Conant et al., 2014). The evolution from subclinical epileptiform activity to clinical epilepsy has been described in several longitudinal case reports, although the timing and severity vary considerably among individuals (Urraca et al., 2013). Consequently, the presence of persistent epileptiform discharges on EEG in clinically seizure-free patients may warrant careful longitudinal neurological follow-up and serial EEG monitoring to facilitate the early recognition and management of epilepsy (Lusk et al., 2016; Conant et al., 2014).

### 4.4 Literature cohort synthesis: clinical implications for genomic medicine

By integrating the present case with a systematic review of ten previously published cases of proximal 15q duplications (Table 1), several clinically relevant observations emerge.

First, the available evidence supports a gene dosage–phenotype relationship in Dup15q syndrome (Al Ageeli et al., 2014). Across the reviewed cases, increasing copy number within the 15q11–q13 region was generally associated with greater cognitive impairment, more severe language deficits, and a higher prevalence of epilepsy, although individual phenotypic variability remained substantial (Urraca et al., 2013; Al Ageeli et al., 2014; Hogart et al., 2010; Lu et al., 2020; Fakir et al., 2026; Bundey et al., 1994; Orrico et al., 2009; Park et al., 2013; Liu et al., 2026; Battaglia et al., 2010).

Second, our literature review suggests that patients with interstitial tetrasomic duplications may exhibit clinical manifestations comparable to those reported in patients with isodicentric chromosome 15 [idic(15)], including severe neurodevelopmental impairment and an increased risk of epilepsy (Urraca et al., 2013; Al Ageeli et al., 2014; Hogart et al., 2010; Lu et al., 2020; Fakir et al., 2026; Bundey et al., 1994; Orrico et al., 2009; Park et al., 2013; Liu et al., 2026; Battaglia et al., 2010). These findings support the concept that overall gene dosage, in addition to chromosomal structure, is an important determinant of phenotypic severity (Al Ageeli et al., 2014). Nevertheless, larger genotype–phenotype studies are required to clarify the relative contributions of copy number, chromosomal architecture, and other genetic or epigenetic modifiers.

Third, our findings underscore the importance of considering an underlying genetic disorder in premature children with persistent and severe neurodevelopmental impairment that cannot be adequately explained by prematurity alone. Because prematurity may obscure the recognition of genetic neurodevelopmental disorders, early genomic evaluation, including CNV analysis or whole-genome sequencing when clinically indicated, may facilitate timely molecular diagnosis and appropriate clinical management (Srivastava et al., 2019).

## 5 Limitations

**FIGURE 4:**
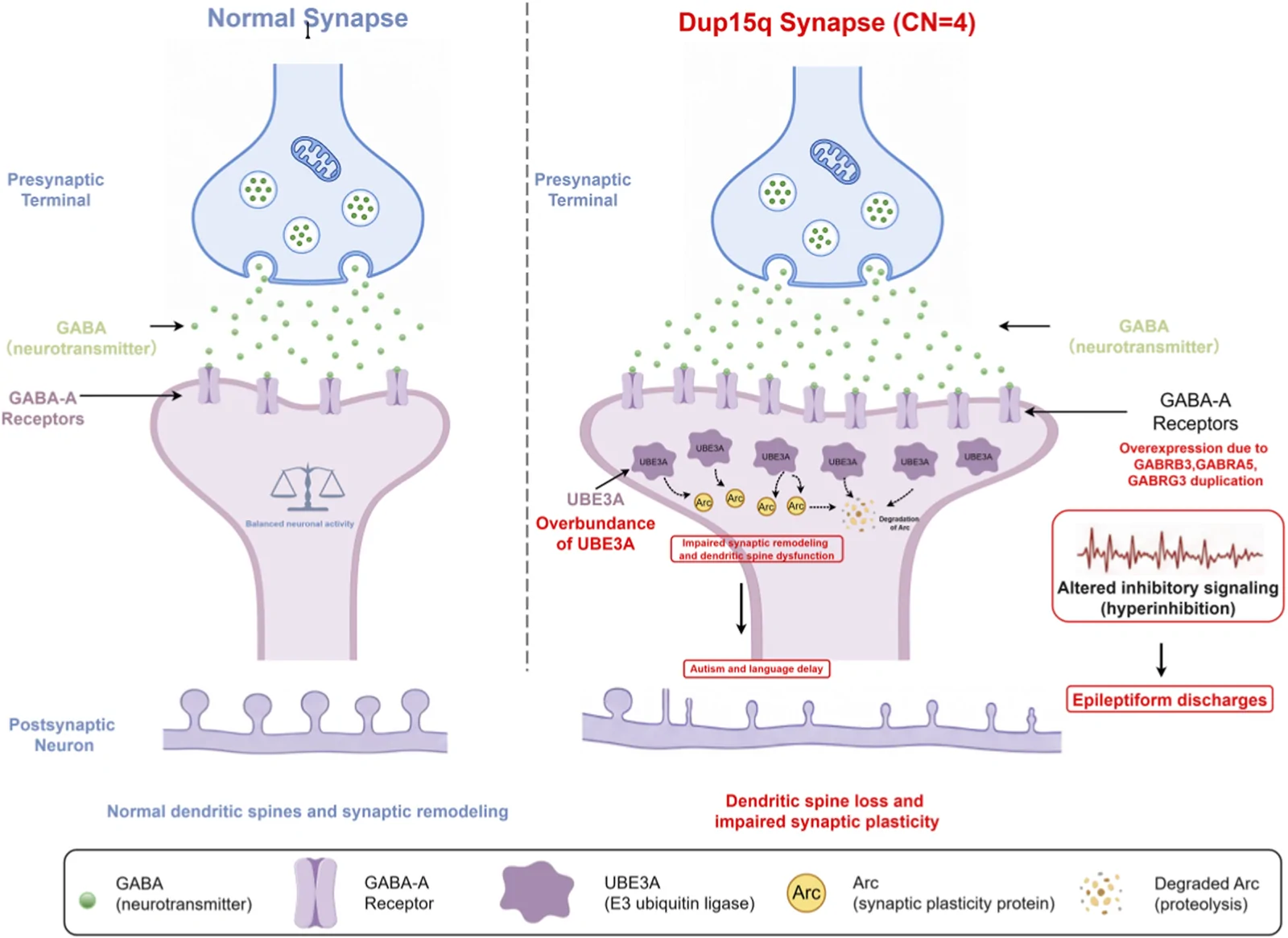
Hypothesized synaptic mechanisms underlying Dup15q syndrome. (Left) In a normal synapse, balanced UBE3A and GABA-A receptor expression maintains healthy dendritic spine density, synaptic remodeling, and homeostatic neuronal activity. (Right) In the Dup15q synapse (CN = 4), two distinct pathogenic pathways drive the clinical phenotype: (1) The GABAergic pathway: Overduplication of the GABRB3, GABRA5, and GABRG3 genes leads to the overexpression of postsynaptic GABA-A receptors, causing altered inhibitory signaling (hyperinhibition) and predisposing the brain to subclinical epileptiform discharges. (2) The UBE3A pathway: Overabundance of the maternally expressed UBE3A (an E3 ubiquitin ligase) results in accelerated proteolysis and degradation of the synaptic plasticity protein Arc. This depletion of Arc impairs synaptic remodeling and leads to dendritic spine loss, representing the neurobiological substrate for autism spectrum disorder (ASD) and language delay.

Several limitations of this study should be acknowledged. First, this is a single retrospective case report, which limits the generalizability of our findings. Second, although we proposed several potential molecular mechanisms to explain the observed clinical phenotype (Figure 4), no functional studies, such as electrophysiological analyses or gene expression assays, were performed to validate these hypotheses. Therefore, the proposed genotype–phenotype relationships remain speculative. Third, although parental CNV analysis confirmed that both duplications had arisen *de novo*, and while high-resolution CNV-seq and WGS established the exact copy number states of the duplicated segments, these technologies are unable to determine the precise physical structure, orientation, or chromosomal configuration (e.g., tandem or insertional) of these rearrangements. Fourth, methylation-specific testing was not available to determine the parental origin of the duplicated chromosome. Finally, long-term clinical follow-up and larger multicenter studies are needed to better define the natural history and genotype–phenotype correlations of this rare genomic rearrangement.

## 6 Conclusion

This report describes a Chinese girl with severe Dup15q syndrome caused by a rare *de novo* complex interstitial duplication comprising a 10.34 Mb tetrasomic duplication of 15q11.1–q13.2 (CN = 4) and an adjacent 2.40 Mb trisomic duplication of 15q13.2–q13.3 (CN = 3). This case expands the molecular and clinical spectrum of complex proximal 15q duplications and highlights the value of high-resolution CNV analysis and whole-genome sequencing in establishing the genetic diagnosis of complex neurodevelopmental disorders.

This case also illustrates the diagnostic challenges posed by prematurity, which may obscure the recognition of an underlying genetic disorder in children with severe neurodevelopmental impairment. Early genomic evaluation should therefore be considered when the severity or progression of neurodevelopmental abnormalities cannot be adequately explained by prematurity alone.

Finally, although conclusions regarding disease mechanisms cannot be drawn from a single case, the present findings support the importance of early multidisciplinary intervention, longitudinal neurological follow-up, and serial EEG assessment in patients with severe Dup15q syndrome to facilitate timely recognition and management of epilepsy.

## Data Availability

The datasets presented in this article are not readily available because of ethical and privacy restrictions. Requests to access the datasets should be directed to the corresponding author.

## References

[B1] Al Ageeli E. Drunat S. Delanoë C. Perrin L. Baumann C. Capri Y. (2014). Duplication of the 15q11-q13 region: clinical and genetic study of 30 new cases. Eur. J. Med. Genet. 57 (1), 5–14. 10.1016/j.ejmg.2013.10.008 24239951

[B2] Battaglia A. (2008). The inv dup (15) or idic (15) syndrome (tetrasomy 15q). Orphanet J. Rare Dis. 3, 30. 10.1186/1750-1172-3-30 19019226 PMC2613132

[B3] Battaglia A. Parrini B. Tancredi R. (2010). The behavioral phenotype of the idic(15) syndrome. Am. J. Med. Genet. A 152A (5), 1104–1110. 10.1002/ajmg.a.33310 20981774

[B4] Blackstone C. (2018). Converging cellular themes for the hereditary spastic paraplegias. Curr. Opin. Neurobiol. 51, 139–146. 10.1016/j.conb.2018.04.025 29753924 PMC6066444

[B5] Bundey S. Hardy C. Vickers S. Kilpatrick M. W. Corbett J. A. (1994). Duplication of the 15q11-13 region in a patient with autism, epilepsy and ataxia. Dev. Med. Child. Neurol. 36 (8), 736–742. 10.1111/j.1469-8749.1994.tb11921.x 8050626

[B6] Conant K. D. Finucane B. Cleary N. Martin A. Muss C. Delany M. (2014). A survey of seizures and current treatments in 15q duplication syndrome. Epilepsia 55 (3), 396–402. 10.1111/epi.12530 24502430

[B7] Christian S. L. Fantes J. A. Mewborn S. K. Huang B. Ledbetter D. H. (1999). Large genomic duplicons map to sites of instability in the prader-Willi/Angelman syndrome chromosome region (15q11-q13). Hum. Mol. Genet. 8 (6), 1025–1037. 10.1093/hmg/8.6.1025 10332034

[B8] Cox D. M. Butler M. G. (2015). The 15q11.2 BP1-BP2 microdeletion syndrome: a review. Int. J. Mol. Sci. 16 (2), 4068–4082. 10.3390/ijms16024068 25689425 PMC4346944

[B9] Fakir Z. Tazzite A. Berrada S. Dehbi H. (2026). Duplication of the 15q11-13 region in a Moroccan patient with autism, intellectual deficiency, and absence of speech. Eur. J. Med. Case Rep. 10, 80–83. 10.24911/ejmcr.9-2401

[B10] Gillentine M. A. Schaaf C. P. (2015). The human clinical phenotypes of altered CHRNA7 copy number. Biochem. Pharmacol. 97 (4), 352–362. 10.1016/j.bcp.2015.06.012 26095975 PMC4600432

[B11] Greer P. L. Hanayama R. Bloodgood B. L. Mardinly A. R. Lipton D. M. Flavell S. W. (2010). The angelman syndrome protein Ube3A regulates synapse development by ubiquitinating Arc. Cell 140 (5), 704–716. 10.1016/j.cell.2010.01.026 20211139 PMC2843143

[B12] Hogart A. Wu D. LaSalle J. M. Schanen N. C. (2010). The comorbidity of autism with the genomic disorders of chromosome 15q11.2-q13. Neurobiol. Dis. 38 (2), 181–191. 10.1016/j.nbd.2008.08.010 18840528 PMC2884398

[B13] Liu R. Y. Wu Y. J. Zou C. C. (2026). Genetic and clinical characteristics of chromosome 15q11-q13 duplication syndrome in Chinese children. Am. J. Med. Genet. A 200 (7), 1498–1514. 10.1002/ajmg.a.63224 41730779

[B14] Lu Y. Liang Y. Ning S. Deng G. Xie Y. Song J. (2020). Rare partial trisomy and tetrasomy of 15q11-q13 associated with developmental delay and autism spectrum disorder. Mol. Cytogenet 13, 21. 10.1186/s13039-020-00489-z 32536972 PMC7288499

[B15] Lusk L. Vogel-Farley V. DiStefano C. Jeste S. (2016). Maternal 15q Duplication Syndrome. Genereviews®. Seattle (WA): University of Washington, Seattle. 27308687

[B16] Orrico A. Zollino M. Galli L. Buoni S. Marangi G. Sorrentino V. (2009). Late-onset Lennox-Gastaut syndrome in a patient with 15q11.2-q13.1 duplication. Am. J. Med. Genet. A 149A (5), 1033–1035. 10.1002/ajmg.a.32785 19396834

[B17] Park D. H. Lim S. Park E. S. Sim E. G. (2013). A nine-month-old boy with isodicentric chromosome 15: a case report. Ann. Rehabil. Med. 37 (2), 291–294. 10.5535/arm.2013.37.2.291 23705128 PMC3660494

[B18] Rainier S. Chai J. H. Tokarz D. Nicholls R. D. Fink J. K. (2003). NIPA1 gene mutations cause autosomal dominant hereditary spastic paraplegia (SPG6). Am. J. Hum. Genet. 73 (4), 967–971. 10.1086/378417 14508710 PMC1180617

[B19] Riggs E. R. Andersen E. F. Cherry A. M. Kantarci S. Kearney H. Patel A. (2020). Technical standards for the interpretation and reporting of constitutional copy-number variants. Genet. Med. 22 (2), 245–257. 10.1038/s41436-019-0686-8 31690835 PMC7313390

[B20] Ross A. J. Sachdev P. S. (2004). Magnetic resonance spectroscopy in cognitive research. Brain Res. Rev. 44 (2-3), 83–102. 10.1016/j.brainresrev.2003.11.001 15003387

[B21] Silva A. I. Haddon J. E. Syed Y. A. Trent S. Lin T. C. E. Patel Y. (2019). Cyfip1 haploinsufficient rats show white matter changes, myelin thinning, abnormal oligodendrocytes and behavioural inflexibility. Nat. Commun. 10, 3455. 10.1038/s41467-019-11119-7 PMC6671959 31371763

[B22] Sinkus M. L. Graw S. Freedman R. Ross R. G. Lester H. A. Leonard S. (2015). The human CHRNA7 and CHRFAM7A genes: a review of the genetics, regulation, and function. Neuropharmacology 96 (Pt B), 274–288. 10.1016/j.neuropharm.2015.02.006 25701707 PMC4486515

[B23] Srivastava S. Love-Nichols J. A. Dies K. A. Ledbetter D. H. Martin C. L. Chung W. K. (2019). Meta-analysis and multidisciplinary consensus statement: exome sequencing is a first-tier clinical diagnostic test for individuals with neurodevelopmental disorders. Genet. Med. 21 (11), 2413–2421. 10.1038/s41436-019-0536-8 31182824 PMC6831729

[B24] Urraca N. Cleary J. Brewer V. Pivnick E. K. McVicar K. Thibert R. L. (2013). The interstitial duplication 15q11.2-q13 syndrome includes autism, mild facial anomalies and a characteristic EEG signature. Autism Res. 6 (4), 268–279. 10.1002/aur.1284 23495136 PMC3884762

[B25] World Medical Association (2013). World medical association declaration of helsinki: ethical principles for medical research involving human subjects. JAMA 310 (20), 2191–2194. 10.1001/jama.2013.281053 24141714

